# 3D shape analyses of extant primate and fossil hominin vertebrae support the ancestral shape hypothesis for intervertebral disc herniation

**DOI:** 10.1186/s12862-019-1550-9

**Published:** 2019-12-16

**Authors:** Kimberly A. Plomp, Keith Dobney, Darlene A. Weston, Una Strand Viðarsdóttir, Mark Collard

**Affiliations:** 1 Department of Archaeology, Classics and Egyptology, University of Liverpool, 14 Abercromby Square, Liverpool, L69 7WZ UK; 2 Department of Archaeology, Simon Fraser University, 8888 University Dr, Burnaby, BC V5A 1S6 Canada; 3 Department of Archaeology, School of Geosciences, University of Aberdeen, St Mary’s, Elphinstone Road, Scotland, UK AB24 3UF Aberdeen,; 4 Department of Anthropology, University of British Columbia, 6303 NW Marine Drive, Vancouver, BC V6T 1Z1 Canada; 5 Biomedical Center, University of Iceland, Læknagarður, Vatnsmýrarvegi 16, 101, Reykjavík, Iceland

**Keywords:** Back pain, Intervertebral disc herniation, Spine, Vertebrae, Bipedalism, Human evolution

## Abstract

**Background:**

Recently we proposed an evolutionary explanation for a spinal pathology that afflicts many people, intervertebral disc herniation (Plomp et al. [2015] *BMC Evolutionary Biology* 15, 68). Using 2D data, we found that the bodies and pedicles of lower vertebrae of pathological humans were more similar in shape to those of chimpanzees than were those of healthy humans. Based on this, we hypothesized that some individuals are more prone to intervertebral disc herniation because their vertebrae exhibit ancestral traits and therefore are less well adapted for the stresses associated with bipedalism. Here, we report a study in which we tested this “Ancestral Shape Hypothesis” with 3D data from the last two thoracic and first lumbar vertebrae of pathological *Homo sapiens*, healthy *H. sapiens*, *Pan troglodytes*, and several extinct hominins.

**Results:**

We found that the pathological and healthy *H. sapiens* vertebrae differed significantly in shape, and that the pathological *H. sapiens* vertebrae were closer in shape to the *P. troglodytes* vertebrae than were the healthy *H. sapiens* vertebrae. Additionally, we found that the pathological human vertebrae were generally more similar in shape to the vertebrae of the extinct hominins than were the healthy *H. sapiens* vertebrae. These results are consistent with the predictions of the Ancestral Shape Hypothesis. Several vertebral traits were associated with disc herniation, including a vertebral body that is both more circular and more ventrally wedged, relatively short pedicles and laminae, relatively long, more cranio-laterally projecting transverse processes*,* and relatively long, cranially-oriented spinous processes. We found that there are biomechanical and comparative anatomical reasons for suspecting that all of these traits are capable of predisposing individuals to intervertebral disc herniation.

**Conclusions:**

The results of the present study add weight to the hypothesis that intervertebral disc herniation in *H. sapiens* is connected with vertebral shape. Specifically, they suggest that individuals whose vertebrae are towards the ancestral end of the range of shape variation within *H. sapiens* have a greater propensity to develop the condition than other individuals. More generally, the study shows that evolutionary thinking has the potential to shed new light on human skeletal pathologies.

## Background

Investigating the causes of back pain is an important undertaking. Up to two-thirds of people experience back pain at some point in their life [1, 2], making it one of the most common medical problems. It is also one of the most serious medical conditions. Surveys indicate that back pain is the single greatest contributor to disability worldwide [3] and this has substantial economic impacts [4]. For example, back pain has been estimated to cost the UK between £3 billion and £12 billion per year [5, 6]. The situation is similar in the US, where it has been calculated that corporations currently lose nearly $7.5 billion per year due to back pain among workers in the 40–65 year age group [7]. Given the individual and societal costs of back pain, there is a pressing need for further research on its causes.

Modern humans are affected by spinal pathologies more often than other, wild-living animals [8, 9] and this has led researchers to propose that our unique mode of posture and locomotion, bipedalism, is one of the factors responsible for back pain [10–15]. Bipedalism, it is argued, places an unusually large amount of stress on the spine that can result in damage to the vertebrae and intervertebral discs [10–15]. While this explanation is intuitively appealing, there has been surprisingly little research on the relationship between spinal pathologies and bipedalism. To date, less than a dozen studies have investigated the topic with data [10–23].

Most of the studies that have examined the relationship between spinal pathologies and bipedalism have focused on spondylolysis, a condition in which a fatigue fracture causes a cleft in the neural arch. Ward et al. [16–18] suggested that susceptibility to spondylolysis is related to an important adaptation for bipedalism, lumbar lordosis, which is a forward curvature of the lower spine. They found that spondylolysis sufferers tend to have reduced medio-lateral spacing between the zygapophyseal facets of adjoining vertebrae, which leads to the articular processes of one vertebra directly contacting the pars interarticularis of the subjacent vertebra, causing the fatigue fracture. In a similar vein, Masharawi et al. [20] discovered that individuals with spondylolysis tend to have more wedge-shaped 5th lumbar vertebrae than unaffected individuals. They suggested that this increases lumbar lordosis and influences the development of spondylolysis by increasing direct contact between the neural arches of the 4th and 5th lumbar vertebrae.

**Fig. 1:**
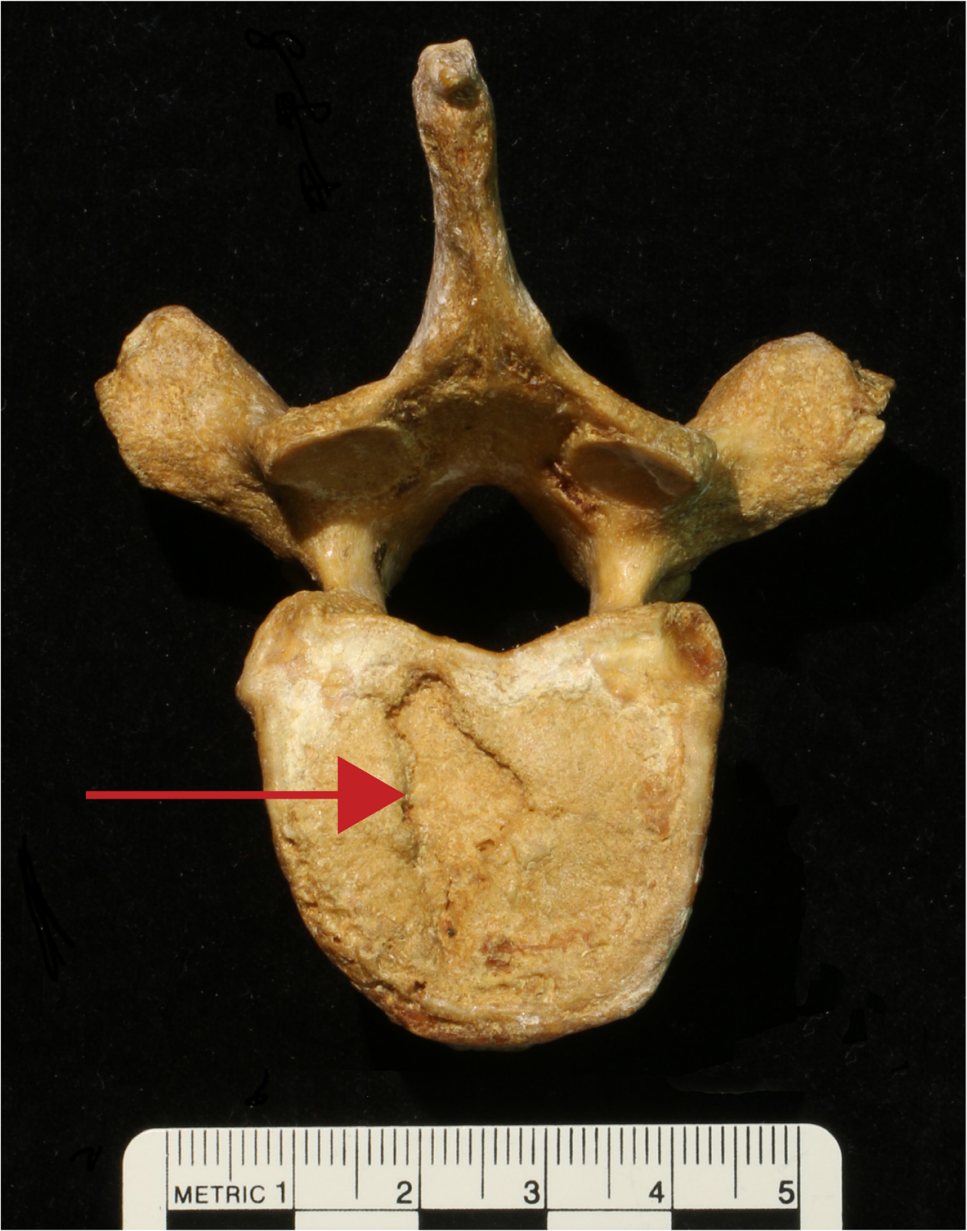
A Schmorl’s node on the inferior endplate of a human thoracic vertebra

Recently we proposed a link between bipedalism and another common spinal pathology, intervertebral disc herniation [15]. Intervertebral disc herniation can be asymptomatic [24–26] or can result in both acute and chronic episodes of back pain [27–29]. One form of it, vertical intervertebral disc herniation, can be recognized on skeletal remains by the presence of Schmorl’s nodes, which are depressions with sclerotic margins on the vertebral endplate (Fig. 1) [30]. We found evidence that *Homo sapiens* vertebrae with Schmorl’s nodes are more similar in shape to the vertebrae of chimpanzees (*Pan troglodytes*) than are healthy *H. sapiens* vertebrae. Because *Homo* and *Pan* share an exclusive common ancestor and there is general agreement that that ancestor was a quadruped (e.g. [31–33]), we proposed that our finding indicated that people who experience intervertebral disc herniation do so because their vertebrae fall at the ancestral end of the range of variation in *H. sapiens* and, therefore, are less well adapted for the stresses associated with bipedalism. We dubbed this the “Ancestral Shape Hypothesis.”

While our previous study and those of Ward and colleagues [16–18] and Masharawi et al. [19, 20] support the hypothesis that there is a relationship between spinal pathologies and bipedalism, and suggest that that relationship is mediated by vertebral shape, further work is required. Most obviously, all of the studies in question relied on two-dimensional data [15–20]. Using such data to analyze three-dimensional (3D) anatomical structures can potentially result in traits being missed or mischaracterized, especially when the structures are complex, as is the case with vertebrae [34]. Thus, an issue that needs to be investigated is whether the findings can be replicated when more detailed, 3D data are employed. With this in mind, we carried out a study in which we used 3D geometric morphometric techniques to test the Ancestral Shape Hypothesis for intervertebral disc herniation [15].

Our study focused on the thoracic and lumbar vertebrae of three extant taxa—*H. sapiens* with Schmorl’s nodes, healthy *H. sapiens*, and *P. troglodytes*—and five extinct hominin taxa—*Australopithecus africanus, Australopithecus sediba*, *Paranthropus robustus*, *Homo naledi*, and *Homo neanderthalensis*. As was the case in our previous study [15], we used the presence of one or more Schmorl’s nodes in an individual’s vertebral column as evidence of intervertebral disc herniation. We carried out three sets of analyses. In the first, we sought to replicate the results of our previous study [15] and so focused on landmarks of the vertebral body, pedicles, and laminae, and compared the pathological *H. sapiens* vertebrae to the healthy *H. sapiens* and *P. troglodytes* vertebrae. In the second of analyses, we again compared the pathological *H. sapiens* vertebrae to the healthy *H. sapiens* and *P. troglodytes* vertebrae, but this time we included landmarks from other parts of the vertebrae, including the spinous and transverse processes, to obtain a more complete picture of the traits associated with intervertebral disc herniation. In the third and final set of analyses, we compared the pathological *H. sapiens* vertebrae not only to the healthy *H. sapiens* and *P. troglodytes* vertebrae, but also to the vertebrae assigned to the five extinct hominin taxa. In order to minimize the contact with the fragile fossil vertebrae, only the landmarks pertaining to the superior part of the vertebrae were used in this set of analyses.

## Methods

We collected data on the penultimate thoracic, final thoracic, and 1st lumbar vertebrae of 132 *H. sapiens* and 35 *P. troglodytes.* Fifty-two of the *H. sapiens* specimens had Schmorl’s nodes in at least one of their vertebrae, while 80 showed no signs of spinal pathology. Schmorl’s nodes were macroscopically diagnosed on the basis of Schmorl and Junghanns’ [24] description. A taxon-by-taxon breakdown of the number of specimens per type of vertebra is given in Table 1. The number of specimens per vertebral type varies within each taxon because some individuals did not preserve all vertebral types. All specimens were determined to be adult on the basis of epiphyseal fusion [35].

**Table 1:** Composition of the extant sample. The number of specimens per vertebra type varies within each taxon because some individuals did not preserve all vertebra types

Taxon	Penultimate thoracic	Final thoracic	First lumbar
Pathological *Homo sapiens*	43	52	37
Healthy *Homo sapiens*	59	66	79
*Pan troglodytes*	33	35	33

We needed to ensure that the specimens of each vertebra type were homologous. To accomplish this, we categorized vertebrae as thoracic or lumbar based on the orientation of the zygapophyseal facets [36, 37]. There were two reasons for using this definition instead of the traditional one in which all vertebra the bear ribs are deemed to be thoracic vertebrae [38–44]. One is that the orientation and curvature of the zygapophyseal facets has been found to be important in posture and locomotion [45–55], making their orientation a critical consideration in this study. The other is that using the “facet definition” allows for the analysis of subtle differences in zygapophyseal shape rather than having the results impacted by the more drastic differences in orientation between thoracic and lumbar facets. In this paper then, “final thoracic vertebra” refers to the diaphragmatic vertebra, which has zygapophyseal joint orientations that are transitional between the thoracic and lumbar spine, with the superior facets exhibiting the thoracic coronal orientation and the lower facets having the lumbar sagittal orientation [46–48]. “Penultimate thoracic” refers to the vertebra that is directly above the diaphragmatic vertebra and has both superior and inferior facets with a thoracic-coronal orientation.

We also collected data on a number of original extinct hominin specimens (Table 2). These included the penultimate thoracic, final thoracic, and 1st lumbar vertebrae of Sts 14, Kebara 2, and Shanidar 3. Sts 14 is a partial skeleton from South Africa that dates to ca. 2.5 Ma and is assigned to *Australopithecus africanus* [56]. Kebara 2 and Shanidar 3 are *H. neanderthalensis* specimens from sites in the Middle East. Kebara 2 is thought to be around 60,000 years old [57], while Shanidar 3 is estimated to be between 35,000 and 65,000 years old [58]. The other extinct hominin specimens we included in our sample are MH 1, SK 3981a, SK 853, and UW 101–1733. MH 1 is a partial skeleton of *Australopithecus sediba* that was recovered at the site of Malapa, South Africa, and is thought to date to 1.9 Ma [59]. SK 3981a and SK 853 are final thoracic vertebra from the site of Swartkrans, South Africa, that date to around 1.8 Ma and have been assigned to *Paranthropus robustus* [56]. UW 101–1733 is a penultimate thoracic vertebra assigned to *Homo naledi*. So far, remains of *H. naledi* have only been found at the site of Rising Star in South Africa. Like most of the other *H. naledi* specimens, UW 101–1733 has been estimated to date to between 236 and 335 Ka [60]. Based on their degree of epiphyseal fusion, Sts 14, Kebara 2, Shanidar 3, SK3981a, and UW 101–1733 were adults when they died, whereas SK 853 and MH1 were juveniles [56–63]. While the inclusion of juvenile specimens introduced another potential source of error, we opted to do so because well-preserved vertebrae are rare in the hominin fossil record and we wished to maximize the size of our sample.

**Table 2:** Fossil specimens included in the present study. See main text for references to support putative locomotor strategy assignments

Specimen	Taxon	Site	Estimated age	Putative locomotor strategy	Preservation	Curation Location
Kebara 2 penultimate thoracic	*Homo neanderthalensis*	Kebara, Israel	60 Ka	Obligate biped	Complete	Tel Aviv University, Israel
Kebara 2 final thoracic	*Homo neanderthalensis*	Kebara, Israel	60 Ka	Obligate biped	Complete	Tel Aviv University, Israel
Kebara 2 first lumbar	*Homo neanderthalensis*	Kebara, Israel	60 Ka	Obligate biped	Complete	Tel Aviv University, Israel
MH 1 first lumbar	*Australopithecus sediba*	Malapa, South Africa	1.9 Ma	Facultative biped	Complete but transverse processes are asymmetric (see text for details of how we dealt with this)	Evolutionary Studies Institute, University of Witwatersrand, Johannesburg, South Africa
Shanidar 3 penultimate thoracic	*Homo neanderthalensis*	Shanidar, Iraq	35–65 Ka	Obligate biped	Complete	Smithsonian National Museum of Natural History, Washington, DC, USA
Shanidar 3 final thoracic	*Homo neanderthalensis*	Shanidar, Iraq	35–65 Ka	Obligate biped	Complete	Smithsonian National Museum of Natural History, Washington, DC, USA
Shanidar 3 first lumbar	*Homo neanderthalensis*	Shanidar,Iraq	35–65 Ka	Obligate biped	Nearly complete but some elements reconstructed	Smithsonian National Museum of Natural History, Washington, DC, USA
SK 853 final thoracic	*Paranthropus robustus*	Swartkrans, South Africa	1.8 Ma	Facultative biped	Mostly complete but missing tip of right transverse process and small portion of right superior zygapophyseal facet	Ditsong National Museum of Natural History, Pretoria, South Africa
SK 3981a final thoracic	*Paranthropus robustus*	Swartkrans, South Africa	1.8 Ma	Facultative biped	Complete with minor damage to body	Ditsong National Museum of Natural History, Pretoria, South Africa
Sts 14 penultimate thoracic	*Australopithecus africanus*	Sterkfontein, South Africa	2.5 Ma	Facultative biped	Complete with minor damage to body	Ditsong National Museum of Natural History, Pretoria, South Africa
Sts 14 final thoracic	*Australopithecus africanus*	Sterkfontein, South Africa	2.5 Ma	Facultative biped	Complete	Ditsong National Museum of Natural History, Pretoria, South Africa
Sts 14 first lumbar	*Australopithecus africanus*	Sterkfontein, South Africa	2.5 Ma	Facultative biped	Minor damage to body and undeveloped left transverse process (see text for details of how we dealt with this)	Ditsong National Museum of Natural History, Pretoria, South Africa
UW 101–1733 penultimate thoracic	*Homo naledi*	Rising Star, South Africa	236–335 Ka	Facultative biped	Nearly complete; missing distal ends of spinous and transverse processes	Evolutionary Studies Institute, University of Witwatersrand, Johannesburg, South Africa

**Fig. 2:**
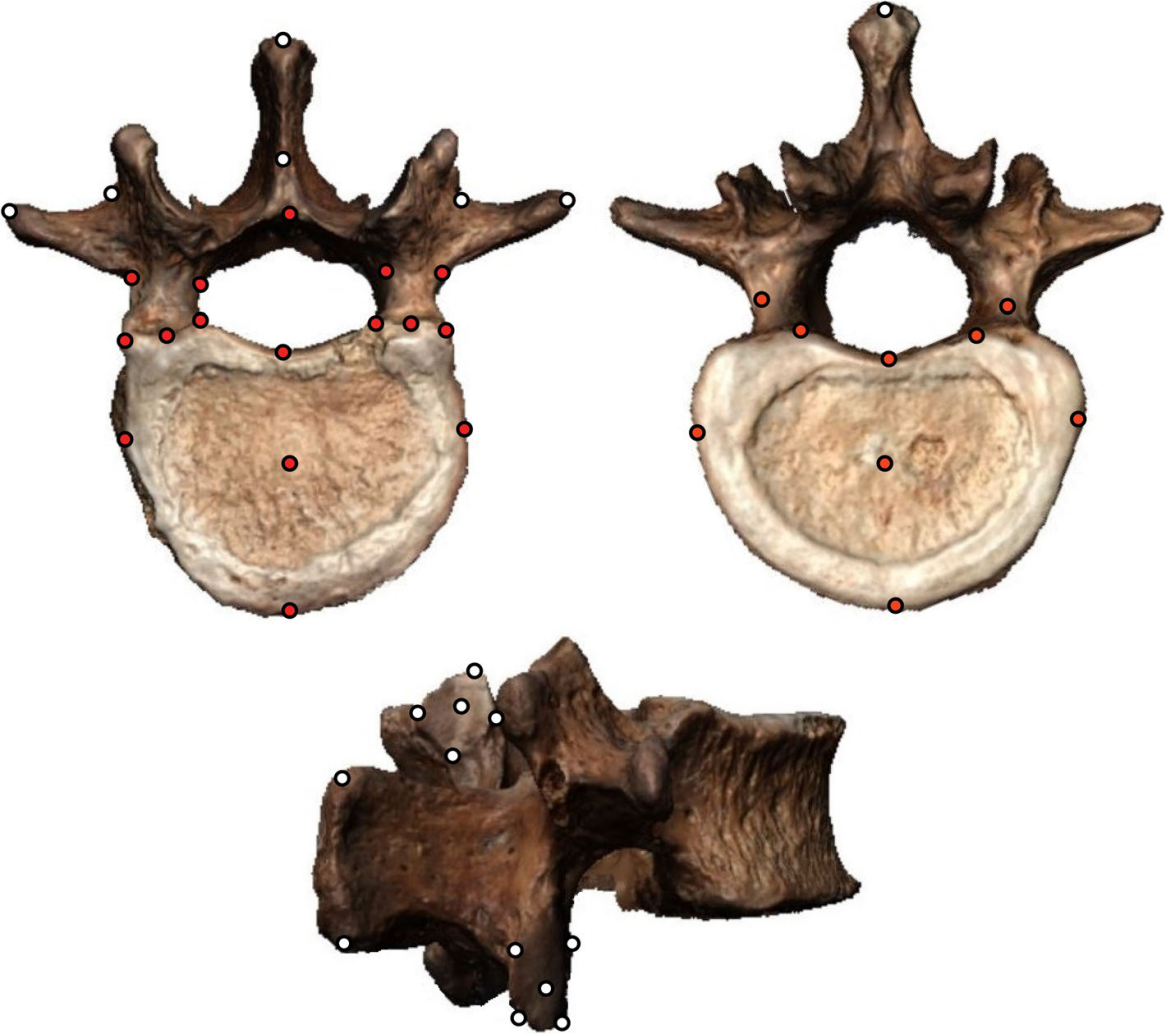
Landmarks used in the analyses. There are 54 in total. The red ones are the 26 that were used in the first set of analyses. In the third set of analyses, the 33 landmarks on the superior surface of the vertebrae were used. The top-left image is the superior view; the top-right image is the inferior view; and the bottom image is the right lateral view

3D coordinates of 54 landmarks were recorded on each extant vertebra by a single observer (KAP) (Fig. 2). The landmarks were chosen to capture the shapes of the body and posterior elements of the vertebrae and included 32 type II and 22 type III landmarks [64]. Landmarks were recorded using a Microscribe digitizing arm. To reduce the effects of recording error, each vertebra was digitized twice and the coordinates averaged [65].

In order to minimize the risk of damage, only 33 of the landmarks were recorded on the fossil specimens (Fig. 2). The landmarks in question capture the shape of the superior surface of the vertebrae and were chosen because they could be obtained with minimal contact with the specimens. Where necessary, missing landmarks were estimated by mirroring the corresponding landmarks from the opposite side (e.g. the landmark on the left transverse process of the first lumbar of Sts 14 and MH1 were reflected to estimate the coordinate of the missing landmark of the right process).

Intra-observer error was assessed as per Neubauer et al. [66, 67]. A single first lumbar vertebra was digitized ten times and then Morphologika [68] was used to compare the greatest Procrustes distance between the ten repeated landmark configurations with the ten smallest Procrustes distances between the landmark configurations of all the first lumbar vertebrae. The smallest distance between the non-repeated vertebrae was almost double the greatest distance between the repeated vertebrae. This amount of landmark recording error is considered unlikely to influence the shape variance of the sample [66, 67].

Having collected and assessed the accuracy of the data, we carried out three sets of analyses. We began by attempting to replicate our previous results [15]. This involved utilizing only the 26 landmarks pertaining to the body, laminae, and pedicles, and comparing just the extant taxa—pathological *H. sapiens*, healthy *H. sapiens*, and *P. troglodytes*.

The first step was to remove the effects of translation, rotation, size, and asymmetry from each dataset independently. This was accomplished by applying the approach outlined by Klingenberg et al. [69] to each dataset in turn. This entailed reflecting and re-labelling the landmark coordinates. Each dataset was then subjected to Generalized Procrustes Analysis (GPA), which is designed to remove translational and rotational effects from landmark data and to scale the configurations to centroid size [70]. Subsequently, asymmetry was removed by calculating the average Procrustes coordinates between the original and reflected landmarks [71, 72]. The GPAs were carried out in Morphologika [68], while the averaging of the Procrustes coordinates was performed in Excel.

The next step was to investigate whether the data were affected by a potentially important confounding factor—size-related shape change or “allometry.” We did so by subjecting each vertebral dataset to a pooled-taxa regression analysis in which the Procrustes coordinates were regressed on the log of centroid size [70, 73]. We found evidence for allometry in all of the 54 landmark datasets (Additional file 1: Table S1). In light of this, we used MorphoJ [74] to regress the Procrustes coordinates on the log of centroid size in order to generate residuals [15, 73]. The residuals were used in all subsequent analyses.

After minimizing allometry in the datasets, we tested to see if sexual dimorphism was a confounding factor. We did so by iteratively subjecting the residuals for the extant penultimate thoracic, final thoracic, and 1st lumbar vertebrae to principal components analyses (PCA) and then running MANOVAs on the PC scores to test for the existence of differences between sexes. The PCAs were performed in Morphologika [68] and the MANOVAs were carried out in SPSS 25.0 [75]. We found that sexual dimorphism did not influence the *P. troglodytes* samples and it was only a significant factor in the first lumbar vertebra of healthy and pathological *H. sapiens* (λ0.518, F = 2.254, *p* = 0.002). Given the limited evidence for sexual dimorphism in vertebral shape in the two taxa, we opted to use pooled-sex datasets in the rest of the analyses.

Once the various potential confounding factors were minimized, we subjected the datasets for the three vertebrae to PCA and Wilks-Lambda MANOVA in order to test predictions of the Ancestral Shape Hypothesis. In order to reduce noise from higher components, we implemented the principal component (PC) reduction procedure outlined by Baylac and Frieβ [76] and Evin et al. [77]. This procedure aims to reduce noise from PCs that account for little variance while retaining all relevant shape information. It tackles this optimization problem by progressively adding PCs into the analyses until the cross-validation percentage (CVP) begins to drop. The PCs that had the highest CVP scores while representing a minimum of 95% of the shape variance were retained for further analyses [76, 77]. The retained PCs were used to calculate the Procrustes distances between the mean shapes of the three groups. Lastly, we applied MANOVAs to the retained PCs to assess the significance of the differences among the taxa. We tested two predictions. One was that there should be a significant difference in shape between the pathological and healthy *H. sapiens* vertebrae. The other was that the pathological *H. sapiens* vertebrae should be closer in shape to the *P. troglodytes* vertebrae than are the healthy *H. sapiens* vertebrae. The PCAs were performed in Morphologika [68], the Procrustes distances were calculated in R [78], and the MANOVAs were carried out in SPSS 25.0 [75].

The second set of analyses was identical to the first set except it used all 54 landmarks rather than just the 26 landmarks pertaining to the body, laminae, and pedicles. The test predictions were also the same as in the first set of analyses.

In the third set of analyses, we included the fossil specimens as well as the extant ones. This necessitated reducing the landmarks to the 33 available for the fossils. The test predictions took into account what is known about the locomotor repertoires of the extinct species. The current consensus is that *A. africanus*, *A. sediba*, *P. robustus*, and *H. naledi* were facultative bipeds that walked on two legs when on the ground but routinely engaged in arboreal climbing, whereas *H. neanderthalensis* was an obligate biped like *H. sapiens* [69, 79–86]. Given this, we tested three predictions. The first was that the pathological *H. sapiens* vertebrae should be closer in shape to the *P. troglodytes* vertebrae than are the healthy *H. sapiens* vertebrae. The second was that the pathological *H. sapiens* vertebrae should be more similar to the *A. africanus*, *A. sediba*, *P. robustus*, and *H. naledi* vertebrae than are the healthy *H. sapiens* vertebrae. The third prediction was that the pathological *H. sapiens* vertebrae should be equally similar to the healthy *H. sapiens* and *H. neanderthalensis* vertebrae. To test these predictions, we used the PC scores to calculate the Procrustes distances between the means of the extant taxa and the individual fossil specimens. As in the previous Procrustes distance analyses, only the PCs that best discriminated between groups and represented a minimum of 95% of the shape variance were utilized, and the Procrustes distances were calculated in R [78].

## Results

### Analyses using extant taxa and 26 landmarks

The results of the first set of analyses are summarized in Table 3. The Procrustes distances and MANOVAs indicate that there are significant shape differences between the pathological and healthy *H. sapiens* vertebrae, which is consistent with the first test prediction. The Procrustes distances and MANOVAs are equally clear-cut with regard to the second test prediction. The Procrustes distances between the pathological *H. sapiens* and the *P. troglodytes* vertebrae are consistently smaller than those between the healthy *H. sapiens* and *P. troglodytes* vertebrae, and all three of the MANOVAs indicate that the mean shape of the pathological *H. sapiens* vertebrae is statistically indistinguishable from the mean shape of the *P. troglodytes* vertebrae. Both of these findings are consistent with the second test prediction. Thus, the results of the first set of analyses are consistent with our previous findings [15] in that they support the Ancestral Shape Hypothesis.

**Table 3:** Results of the first set of analyses. Procrustes distances and MANOVAs were used to compare pathological *H. sapiens* vertebrae with healthy *H. sapiens* and *P. troglodytes* vertebrae. Procrustes distances were generated from PCs that accounted for ≥95% of the shape variance. These PCs were also used in the MANOVAs. Analyses are grouped on the basis of vertebral type and arranged by the types’ position in the vertebral column. PCs = Number of retained PCs plus the percentage of shape variance they explain. PD = Procrustes distance

Vertebra	PCs	Comparison	PD	MANOVA
Penultimate thoracic	1–23 (94.9%)	Pathological *H. sapiens* vs Healthy *H. sapiens* Pathological *H. sapiens* vs *P. troglodytes* Healthy *H. sapiens* vs *P. troglodytes*	0.0319 0.0207 0.0479	λ 0.593, F = 2.476, *p* = 0.001* λ 0.788, F = 0.645, *p* = 0.876 λ 0.411, F = 4.361, *p* < 0.0001*
Final Thoracic	1–39 (94.9%)	Pathological *H. sapiens* vs Healthy *H. sapiens* Pathological *H. sapiens* vs *P. troglodytes* Healthy *H. sapiens* vs *P. troglodytes*	0.0295 0.0250 0.0401	λ 0.554, F = 1.610, *p* = 0.038* λ 0.640, F = 0.677, *p* = 0.984 λ 0.453, F = 1.889, *p* = 0.013*
First Lumbar	1–24 (94.6%)	Pathological *H. sapiens* vs Healthy *H. sapiens* Pathological *H. sapiens* vs *P. troglodytes* Healthy *H. sapiens* vs *P. troglodytes*	0.0247 0.0305 0.0474	λ 0.536, F = 1.753, *p* = 0.020* λ 0.518, F = 7.29, *p* = 0.819 λ 0.466, F = 2.295, *p* < 0.001*

### Analyses using extant taxa and all landmarks

Table 4 summarizes the results of the second set of analyses. The Procrustes distances indicate that there are shape differences between the pathological and healthy *H. sapiens* vertebrae, while the MANOVAs indicate that the differences are statistically significant. This is consistent with the first test prediction. The analyses also support the second test prediction. Not only were the Procrustes distances between the pathological *H. sapiens* and *P. troglodytes* vertebrae smaller than those between healthy *H. sapiens* and *P. troglodytes* in all three vertebrae, but also the MANOVAs indicated that there was not a significant shape difference between the penultimate and final thoracic vertebrae of pathological *H. sapiens* and *P. troglodytes*. As for the first lumbar vertebrae, the MANOVA comparing the pathological *H. sapiens* and *P. troglodytes* vertebrae returned a significant result.

**Table 4:** Results of the second set of analyses. Procrustes distances and MANOVAs were used to compare pathological *H. sapiens* vertebrae with healthy *H. sapiens* and *P. troglodytes* vertebrae. Procrustes distances were generated from the PCs that accounted for ≥95% of the shape variance. Those PCs were also used in the MANOVAs. Analyses are grouped on the basis of vertebral type and arranged by the types’ position in the vertebral column. PCs = Number of retained PCs plus the percentage of shape variance they explain. PD = Procrustes distance

Vertebra	PCs	Comparison	PD	MANOVA
Penultimate Thoracic	1–36 (95.0%)	Pathological *H. sapiens* vs Healthy *H. sapiens* Pathological *H. sapiens* vs *P. troglodytes* Healthy *H. sapiens* vs *P. troglodytes*	0.0320 0.0473 0.0708	λ 0.509, F = 1.850, *p* = 0.014* λ 0.609, F = 0.570, *p* = 0.810 λ 0.314, F = 3.404, *p* < 0.0001***
Final thoracic	1–38, (95.1%)	Pathological *H. sapiens* vs Healthy *H. sapiens* Pathological *H. sapiens* vs *P. troglodytes* Healthy *H. sapiens* vs *P. troglodytes*	0.0218 0.0478 0.0540	λ 0.506, F = 2.027, *p* = 0.004* λ 0.482, F = 1.355, *p* = 0.159 λ 0.387, F = 2.586, p < 0.0001***
First Lumbar	1–36 (95.2%)	Pathological *H. sapiens* vs Healthy *H. sapiens* Pathological *H. sapiens* vs *P. troglodytes* Healthy *H. sapiens* vs *P. troglodytes*	0.0290 0.0682 0.0813	λ 0.548, F = 1.741, *p* = 0.022* λ 0.248, F = 2.525, *p* = 0.006* λ 0.399, F = 3.146, *p* < 0.0001*

**Fig. 3:**
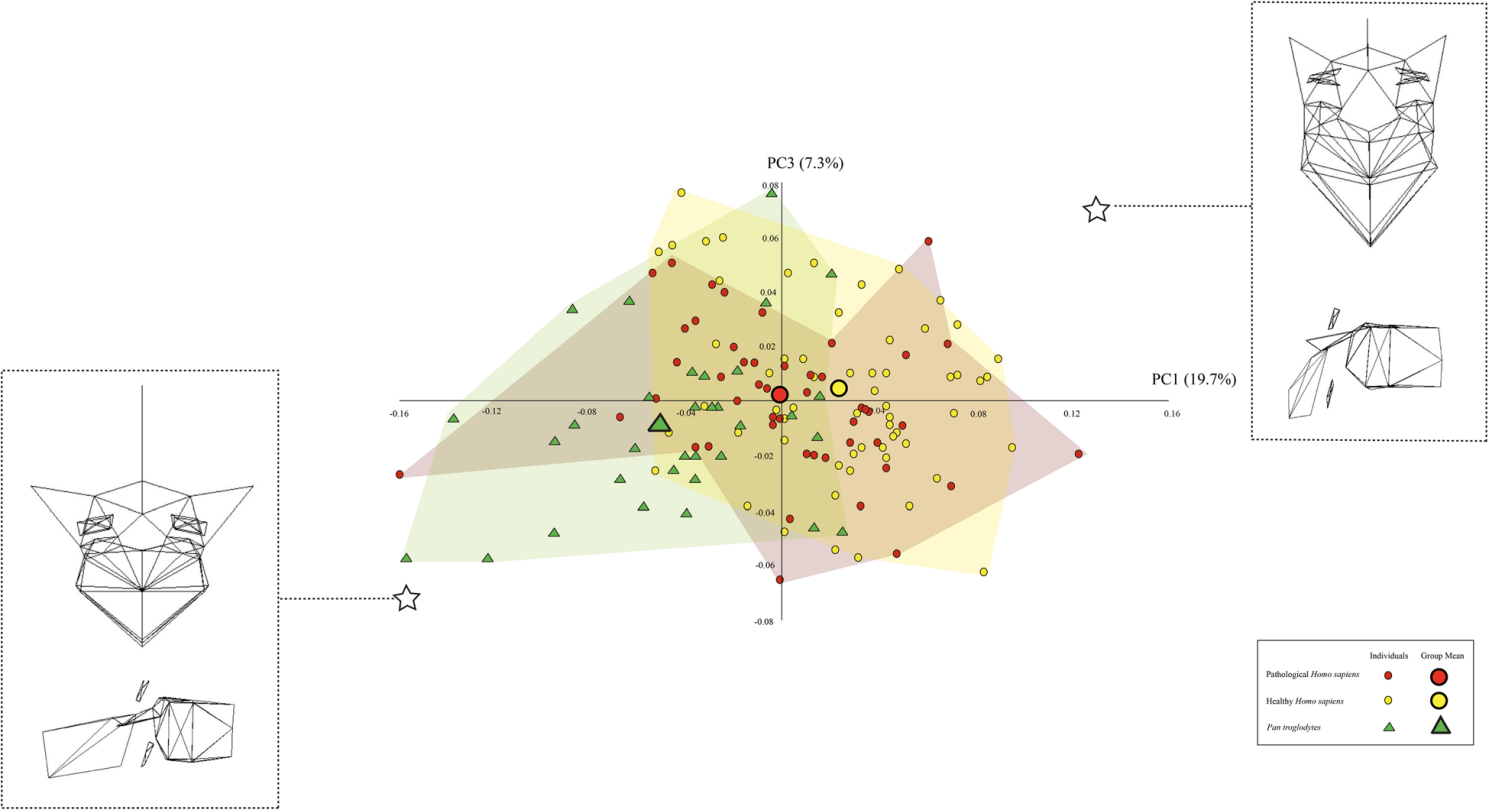
Shape variation in the extant penultimate thoracic vertebrae captured by PCs 1 and 3, which account for 19.7 and 7.3% of the variation, respectively. PC2 did not did not reveal differences among the taxa and therefore was replaced with PC3. The wireframes illustrate the vertebral shapes described by PC1 and PC3. The stars indicate where the wireframes are located in the scatter-plot

**Fig. 4:**
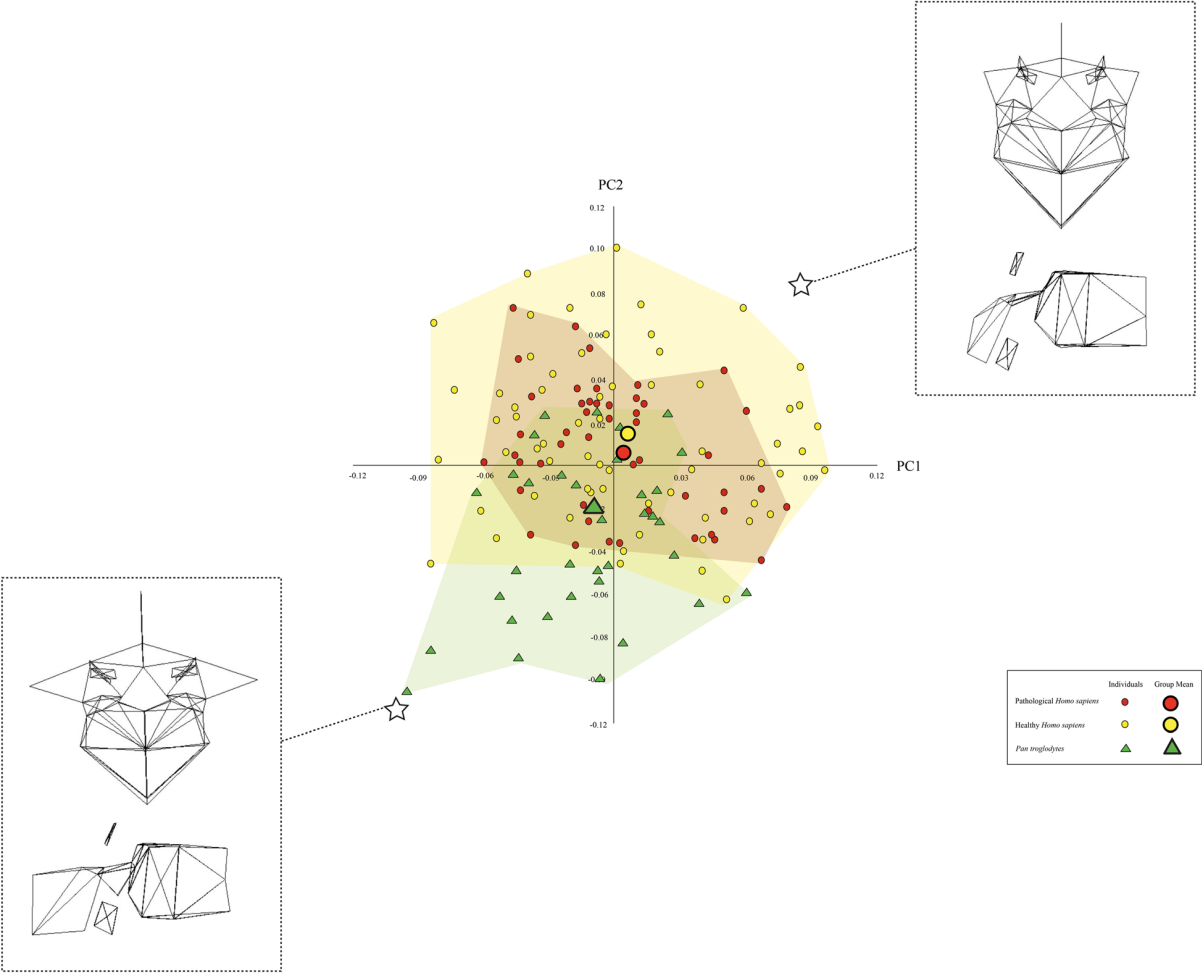
Shape variation in the extant final thoracic vertebrae captured by PCs 1 and 2, which account for 15 and 12.7% of the variation, respectively. The wireframes illustrate the vertebral shapes described by PC1 and PC2. The stars indicate where the wireframes are located in the scatter-plot

**Fig. 5:**
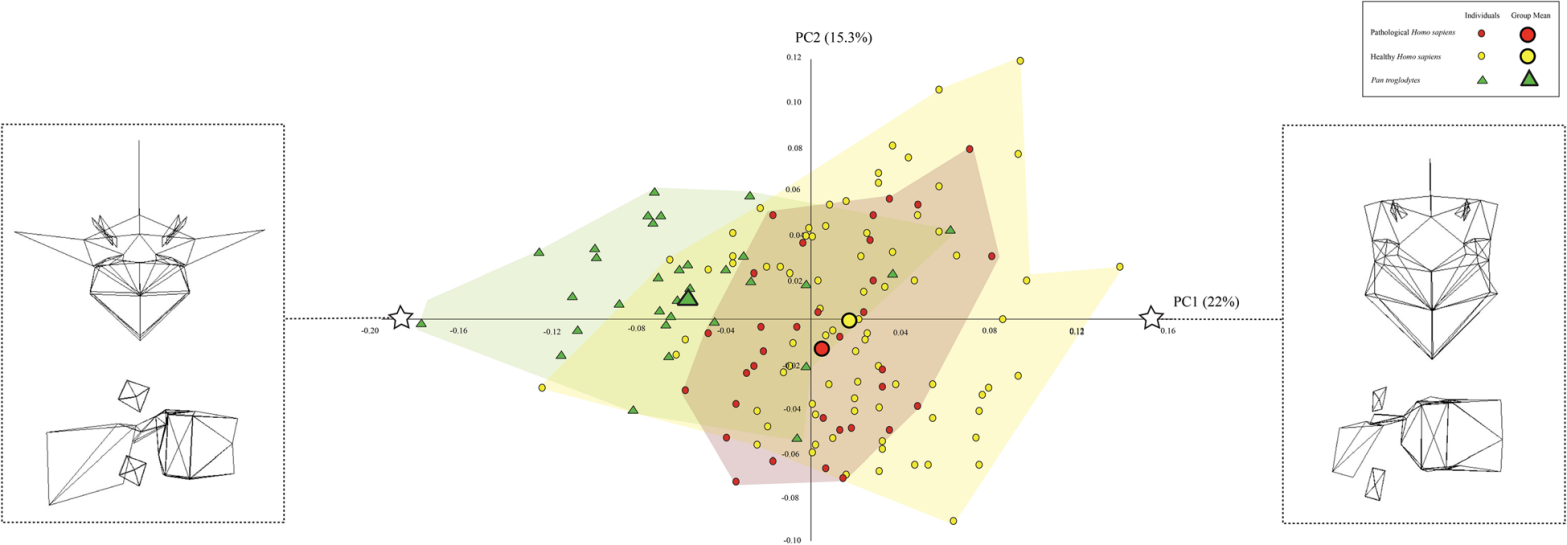
Shape variation in the extant first lumbar vertebrae captured by PCs 1 and 2, which account for 22.0% and 15.3% of the variation, respectively. The wireframes illustrate the vertebral shapes described by PC1. The stars indicate where the wireframes are located in the scatter-plot

Figures 3, 4, and 5 illustrate the similarities and differences among the three taxa on the basis of the most informative PCs. In each scatter plot, the centre of the distribution of the pathological *H. sapiens* specimens tends to be located between the centres of the distributions of the healthy *H. sapiens* and *P. troglodytes* specimens. The wireframes show that there are commonalities among the three vertebral types in the way that pathological *H. sapiens* vertebrae differ from healthy *H. sapiens vertebrae*. Compared to healthy *H. sapiens* vertebrae, pathological *H. sapiens* vertebrae have shorter pedicles and laminae and smaller vertebral foramina. They also have bodies that are more ventrally wedged and circular in planform. In addition, the transverse processes are longer and project more in both the cranial and lateral directions. Lastly, the spinous processes are longer, more cranially oriented, and have cranio-caudally taller tips. Importantly for present purposes, these traits also differentiate the *P. troglodytes* specimens from the healthy *H. sapiens* vertebrae.

### Analyses using fossil and extant taxa and 33 landmarks

Table 5 summarizes the results of the third set of analyses. The comparisons involving just the extant taxa are consistent with the first test prediction. In all cases, the pathological *H. sapiens* vertebrae are closer to the *P. troglodytes* vertebrae than are the healthy *H. sapiens* vertebrae.

**Table 5:** Results of the third set of analyses. Procrustes distances were used to compare the mean of the sample of pathological *H. sapiens* vertebrae with the mean shapes of the healthy *H. sapiens* and *P. troglodytes* vertebrae samples, and with the fossil hominin vertebra. Analyses are grouped on the basis of the fossil specimen they included and are arranged in natural sort order and then by position in the vertebral column. PCs = Number of retained PCs plus the percentage of shape variance they explain. PD = Procrustes distance

Fossil specimen (species)	PCs	Comparison	PD
Kebara 2 penultimate thoracic (*Homo neanderthalensis*)	PCs 1–25 (95.2%)	Pathological *H. sapiens* vs *P. troglodytes* Healthy *H. sapiens* vs *P. troglodytes* Pathological *H. sapiens* vs Kebara 2 Healthy *H. sapiens* vs Kebara 2	0.0879 0.1047 0.0179 0.0283
Kebara 2 final thoracic (*Homo neanderthalensis*)	PCs 1–42 (95.1%)	Pathological *H. sapiens* vs *P. troglodytes* Healthy *H. sapiens* vs *P. troglodytes* Pathological *H. sapiens* vs Kebara 2 Healthy *H. sapiens* vs Kebara 2	0.0681 0.0729 0.0222 0.0226
Kebara 2 first lumbar (*Homo neanderthalensis*)	PCs 1–38 (95.2%)	Pathological *H. sapiens* vs *P. troglodytes* Healthy *H. sapiens* vs *P. troglodytes* Pathological *H. sapiens* vs Kebara 2 Healthy *H. sapiens* vs Kebara 2	0.1098 0.1134 0.0442 0.0552
MH1 first lumbar (*Australopithecus sediba*)	PCs 1–38 (95.2%)	Pathological *H. sapiens* vs *P. troglodytes* Healthy *H. sapiens* vs *P. troglodytes* Pathological *H. sapiens* vs MH1 Healthy *H. sapiens* vs MH1	0.1098 0.1134 0.0451 0.0561
Shanidar 3 penultimate thoracic (*Homo neanderthalensis*)	PCs 1–25(95.2%)	Pathological *H. sapiens* vs *P. troglodytes* Healthy *H. sapiens* vs *P. troglodytes* Pathological *H. sapiens* vs Shanidar 3 Healthy *H. sapiens* vs Shanidar 3	0.0879 0.1047 0.0269 0.0329
Shanidar 3 final thoracic (*Homo neanderthalensis*)	PCs 1–42 (95.1%)	Pathological *H. sapiens* vs *P. troglodytes* Healthy *H. sapiens* vs *P. troglodytes* Pathological *H. sapiens* vs Shanidar 3 Healthy *H. sapiens* vs Shanidar 3	0.0681 0.0729 0.0188 0.0202
Shanidar 3 first lumbar (*Homo neanderthalensis*)	PCs 1–38 (95.2%)	Pathological *H. sapiens* vs *P. troglodytes* Healthy *H. sapiens* vs *P. troglodytes* Pathological *H. sapiens* vs Shanidar 3 Healthy *H. sapiens* vs Shanidar 3	0.1098 0.1134 0.0365 0.0468
SK853 final thoracic (*Paranthropus robustus*)	PCs 1–42 (95.1%)	Pathological *H. sapiens* vs *P. troglodytes* Healthy *H. sapiens* vs *P. troglodytes* Pathological *H. sapiens* vs SK853 Healthy *H. sapiens* vs SK853	0.0681 0.0729 0.0377 0.0404
SK3981a final thoracic (*Paranthropus robustus*)	PCs 1–42 (95.1%)	Pathological *H. sapiens* vs *P. troglodytes* Healthy *H. sapiens* vs *P. troglodytes* Pathological *H. sapiens* vs SK3981a Healthy *H. sapiens* vs SK3981a	0.0681 0.0729 0.0464 0.0450
Sts-14 penultimate thoracic (*Australopithecus africanus*)	PCs 1–40 (95.1%)	Pathological *H. sapiens* vs *P. troglodytes* Healthy *H. sapiens* vs *P. troglodytes* Pathological *H. sapiens* vs Sts-14 Healthy *H. sapiens* vs Sts-14	0.0879 0.1047 0.0435 0.0453
Sts-14 final thoracic (*Australopithecus africanus*)	PCs 1–42 (95.1%)	Pathological *H. sapiens* vs *P. troglodytes* Healthy *H. sapiens* vs *P. troglodytes* Pathological *H. sapiens* vs Sts-14 Healthy *H. sapiens* vs Sts-14	0.0681 0.0729 0.0244 0.0270
Sts-14 first lumbar (*Australopithecus africanus*)	PCs 1–38 (95.2%)	Pathological *H. sapiens* vs *P. troglodytes* Healthy *H. sapiens* vs *P. troglodytes* Pathological *H. sapiens* vs Sts-14 Healthy *H. sapiens* vs Sts-14	0.1098 0.1134 0.0300 0.0388
U.W. 101–1733 penultimate thoracic (*Homo naledi*)	PCs 1–25 (95.2%)	Pathological *H. sapiens* vs *P. troglodytes* Healthy *H. sapiens* vs *P. troglodytes* Pathological *H. sapiens* vs U.W. 101–1733 Healthy *H. sapiens* vs U.W. 101–1733	0.0879 0.1047 0.0138 0.0287

The comparisons involving the fossil specimens are less straightforward with respect to the relevant test predictions. To reiterate, there were two of them. One was that the pathological *H. sapiens* should be closer to *A. africanus*, *A. sediba*, *P. robustus*, and *H. naledi* than are healthy *H. sapiens*. This prediction was supported by all but one of the relevant comparisons. The exception was the comparison involving the *P. robustus* specimen SK 3981a, which indicated that the shape difference between pathological *H. sapiens* and SK 3981a is greater than the shape difference between healthy *H. sapiens* and SK 3981a.

The other prediction involving the fossil hominin specimens was that the pathological *H. sapiens* vertebrae should be equally similar to the healthy *H. sapiens* and *H. neanderthalensis* vertebrae. This prediction was not supported by any of the relevant comparisons. In all six of the analyses that involved the *H. neanderthalensis* specimens, the pathological *H. sapiens* vertebrae were more similar to the *H. neanderthalensis* vertebrae than were the healthy *H. sapiens* vertebrae.

## Discussion and conclusions

The present paper reports three sets of analyses designed to evaluate the Ancestral Shape Hypothesis for intervertebral disc herniation, which contends that individuals whose vertebrae are towards the ancestral end of the range of shape variation within *H. sapiens* have a greater propensity to develop the condition than other individuals. In the first set of analyses, we found that the bodies, pedicles, and laminae of the lower thoracic and upper lumbar vertebrae of pathological and healthy *H. sapiens* differ significantly in shape, and that the differences are such that pathological *H. sapiens* vertebrae are closer in shape to *P. troglodytes* vertebrae than are healthy *H. sapiens* vertebrae. The second set of analyses, which included data from additional parts of the vertebrae, also showed that pathological *H. sapiens* vertebrae are closer in shape to *P. troglodytes* vertebrae than are healthy *H. sapiens* vertebrae. The final set of analyses indicated that pathological *H. sapiens* vertebrae are generally closer in shape to the vertebrae of a number of extinct hominin specimens than are healthy *H. sapiens* vertebrae. Together, these findings suggest that the vertebrae of people who suffer from intervertebral disc herniation tend to fall at the ancestral end of the range of shape variation within *H. sapiens*. This supports the Ancestral Shape Hypothesis [15].

In a previous study, we found that compared to healthy *H. sapiens* vertebrae, pathological *H. sapiens* and *P. troglodytes* vertebrae have relatively smaller neural foramina, shorter, wider pedicles, and rounder vertebral bodies [15]. The 3D data reported here support the existence of these differences and reveal some additional ones, especially in the thoracic vertebrae. In both thoracic and the first lumbar, we found that the vertebral bodies of pathological *H. sapiens* and *P. troglodytes* vertebrae are more ventrally wedged than healthy *H. sapiens* vertebrae. In addition, compared to healthy *H. sapiens* vertebrae, the thoracic vertebrae of pathological *H. sapiens* and *P. troglodytes* vertebrae tend to have longer transverse processes that project more in both the cranial and lateral directions, and longer spinous processes that are more cranially oriented and have cranio-caudally taller tips.

Of the additional putative ancestral traits, perhaps the most noteworthy is the increased ventral wedging of the first lumbar vertebra relative to those of healthy *H. sapiens*. Greater ventral wedging in lumbar vertebrae can be expected to result in a smaller lumbar lordosis angle, i.e. a straighter lower back [87]. Hence, our results indicate that people who are prone to intervertebral disc herniation tend to have a straighter back than unaffected *H. sapiens* and suggest that this is an ancestral trait. Both of these hypotheses are supported by the available data on lumbar lordosis angle in *H. sapiens*, *P. troglodytes*, and extinct hominins. The average lumbar lordosis angle for healthy humans is 51° [88]. Few data on *P. troglodytes* are available but those we have suggest that chimpanzees have a lumbar lordosis angle of around 22° [89]. Recently, Been et al. [88, 90–92] and Gomez-Olivencia et al. [93] estimated the lumbar lordosis angle of the *A. africanus* individual Sts-14 and several Neanderthal specimens. They found that Sts-14 would have had a lumbar lordosis angle of 43°, while the average they calculated for the Neanderthal specimens is 29°. Haeusler et al. [94] have also recently reconstructed the lordosis angle of a Neanderthal specimen, La Chapelle-aux-Saints 1. They found that its lordosis angle is 52°, which is close to the average of healthy *H. sapiens*. The differences in estimated lumbar lordosis angles for *H. neanderthalensis* may indicate that the extent of lordosis varied considerably in Neanderthals. Alternatively, it may be the case that the difference in methodology between the studies caused error in one or more reconstructions. Significantly for present purposes, modern humans with intervertebral disc hernias have been found to have an average lumbar lordosis angle of 37° [95, 96]. Thus, the pathological *H. sapiens* value not only falls between those for healthy *H. sapiens* and *P. troglodytes*, it lies closer to Been et al.’s [88, 90–92] and Gomez-Olivencia et al.’s [93] estimates for the australopiths and *H. neanderthalensis* than to the value for healthy *H. sapiens*.

**Fig. 6:**
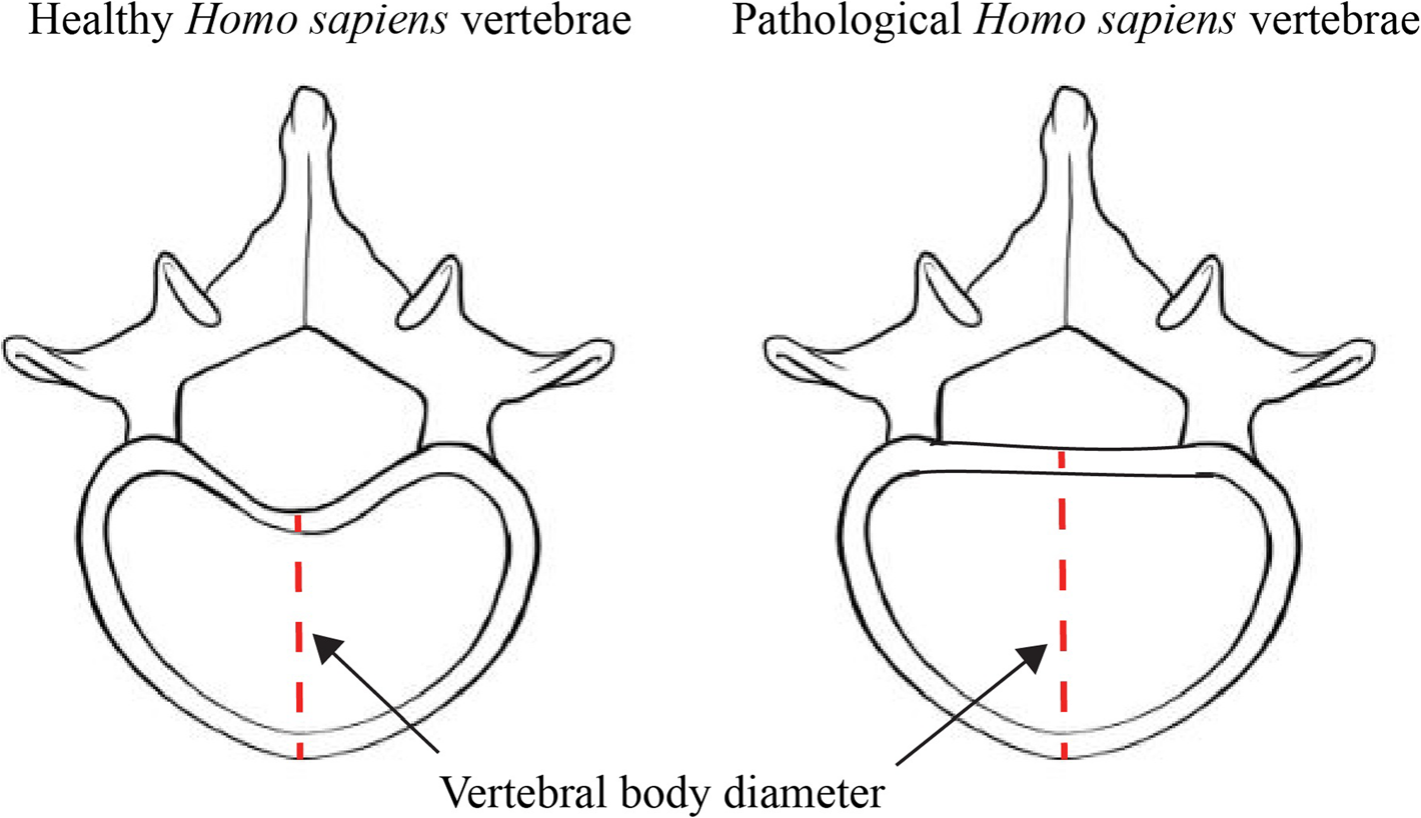
Cartoon illustrating the differences in diameter between a heart-shaped vertebral body and a more circular vertebral body

Why might the putative ancestral traits predispose individuals to develop intervertebral disc herniation? As we noted in our 2015 paper [15], a possible functional explanation for the association between intervertebral disc herniation and vertebral shape is provided by Harrington et al. [97]. These authors suggest that the diameter of the vertebral disc influences its ability to withstand tension during compression. Their argument is based on LaPlace’s law [98], which states that the ability of a fluid-filled tube to withstand tension decreases with increasing radius. According to Harrington et al. [97], the rounder bodies of pathological vertebrae would have larger diameters than the more heart-shaped bodies seen in healthy vertebrae, making the intervertebral disc less able to withstand stress (Fig. 6) [15, 98, 99].

We explained earlier that the more pronounced ventral wedging of the first lumbar vertebrae of pathological humans would result in a lumbar spine with a smaller lumbar lordosis angle [87], and showed that this is supported by studies in which lumbar lordosis angle has been measured in living humans [95, 96]. The authors of the latter studies assumed that the smaller lumbar lordosis angle of the pathological individuals was a consequence of disc degeneration, especially loss of disc height [95, 96]. Our findings challenge this idea by suggesting that the small lumbar lordosis angle in pathological individuals is connected with wedging of the ventral body and therefore is present before herniation. If this is the case, then it is possible that a relatively low lumbar lordosis angle predisposes an individual to intervertebral disc hernias. A potential biomechanical explanation for this is that lumbar lordosis is thought to absorb the compressive loads acting on the spine during bipedalism [47, 100, 101]. Thus, a smaller lumbar lordosis angle may decrease the ability of the lower spine to withstand compressive loads.

The shape of the posterior vertebral elements may also influence an individual’s susceptibility to intervertebral disc herniation. To reiterate, we found that compared to healthy *H. sapiens* vertebrae, pathological *H. sapiens* vertebrae tended to have shorter pedicles and laminae. The pedicles and laminae act as structural buttresses for the vertebral body and play an important role in load bearing during axial compression [102–104], and it has been hypothesized that the shorter pedicles and laminae identified in vertebrae with Schmorl’s nodes may be less able to adequately buttress these loads than the longer pedicles and laminae of healthy human vertebrae [15, 99, 105].

In addition, we found that pathological thoracic vertebrae tended to have relatively longer, cranially-oriented spinous processes and longer, cranio-laterally projecting transverse processes. Comparative analyses suggest that the length and orientation of spinous processes relate to spinal mobility and stability [46, 106–110]. Long, cranially-oriented spinous processes are found in many arboreal monkey species and are thought to allow for a greater amount of dorsal mobility in the spine, while short, caudally oriented spinous processes are associated with a less mobile, more stable spine [46, 93, 110–114]. Similarly, mediolaterally longer transverse processes would allow for lateral flexion in the lower spine [46, 112, 113], and transverse processes that project more laterally may be less able to maintain lumbar lordosis than those that project dorsally [43, 46, 88, 92, 93, 112]. Together, these observations suggest that the longer, cranially oriented spinous processes and longer, laterally projecting transverse processes of vertebrae with Schmorl’s nodes may increase the dorsal mobility of the spine compared to shorter, cranially/cranially-dorsally oriented processes of healthy vertebrae. This in turn implies that the traits in question may not provide adequate stability during bipedalism, and - in combination with the short pedicles and laminae and circular, ventrally wedged vertebral bodies - may predispose individuals to intervertebral disc hernias.

That the two *P. robustus* final thoracic vertebrae, SK 853 and SK 3981a, yielded conflicting results was unexpected. To reiterate, the comparison that involved SK 853 supported the relevant test prediction while the comparison that involved SK 3981a did not. Given that the specimens are assigned to the same species, it is surprising that they yielded conflicting results. There are three obvious potential explanations for this. One is that, even though SK 3981a appeared normal to us and to the specimen’s original describer [56], it is either pathological or deformed and, therefore, is more similar to healthy *H. sapiens* vertebrae than it should be. The second possibility is that SK 853 and SK 3981a yielded different results because SK 853 is a juvenile specimen. Under this hypothesis, the developmental status of SK 853 caused it to be misleadingly similar to the other early hominin specimens in the sample and to the pathological *H. sapiens* vertebrae. The third possibility is that SK 3981a and SK 853 do not in fact belong to the same species. Ascertaining which of these hypotheses is most likely to be correct will require further research.

The results of the analyses featuring the Neanderthal specimens were also unexpected. We predicted that the pathological *H. sapiens* vertebrae would be intermediate between the *H. neanderthalensis* and healthy *H. sapiens* vertebrae, but the pathological *H. sapiens* vertebrae were in fact closer to the *H. neanderthalensis* vertebrae than to the healthy *H. sapiens* vertebrae. Given that the analyses in question involved six different Neanderthal specimens, we think it is unlikely that either unrecognized pathology or deformation caused the analyses to fail to support the prediction. The only other obvious explanation is that the test prediction was wrong. As we explained earlier, we based the prediction on the fact that it is widely accepted that Neanderthals were obligate bipeds like modern humans. It seemed reasonable to suppose therefore that Neanderthal vertebrae should have similar bipedalism-related adaptations as modern human vertebrae. However, the analyses suggest that this is not the case. In all the relevant analyses, the distance between the pathological *H. sapiens* mean and the *H. neanderthalensis* mean was smaller than the distance between the healthy *H. sapiens* mean and the *H. neanderthalensis* mean. Given the results of the first two sets of analyses, this not only indicates that Neanderthal lower thoracic and lumbar vertebrae are not identical to their healthy modern human counterparts, it also suggests that they have a number of plesiomorphic features. This raises the possibility that Neanderthals may have been prone to intervertebral disc herniation. Interestingly, Haeusler [115] has recently reported that the La Chapelle-aux-Saints 1 Neanderthal specimen has Schmorl’s nodes in its eighth, ninth, and tenth thoracic vertebrae.

With regard to future research, three tasks suggest themselves. The first and most obvious is to test the foregoing biomechanical hypotheses linking the ancestral traits with propensity to develop intervertebral disc herniation. This could be accomplished by using medical imaging technology and 3D morphometrics to investigate the interaction between bipedalism, vertebral shape, and the soft tissues of the spine in pathological and healthy humans.

It would also be useful to perform analyses similar to the current one on individuals with other spinal pathologies. Spondylolysis - a cleft in the neural arch caused by a fatigue fracture at the site of the pars interarticularis [116] – would be an obvious next target because it has been linked to both bipedalism and vertebral morphology [16]. Specifically, it would be interesting to explore how those vertebral traits associated with spondylolysis identified by Ward and colleagues [16–19] and Masharawi et al. [19, 20] relate to *H. sapiens* and non-human ape vertebral variation. The findings of such a study could provide important information to help researchers and clinicians understand how and why posture and locomotion can influence traumatic conditions like spondylolysis.

Lastly, it would be helpful to test the most basic of the assumptions made by the Ancestral Shape Hypothesis, which is that the causal arrow goes from vertebral shape to intervertebral disc herniation rather than vice versa. There are reasons to believe this is the case. Most notably, the shape of the vertebral foramen is known not to change once the neural arch fuses to the vertebral body [115, 116]. This implies that the pedicles, laminae, and vertebral body, which form the vertebral foramen, also do not change shape once the neural arch fuses to the vertebral body. However, the assumption still needs to be tested. It seems likely that doing so will require a longitudinal study.

## Supplementary information

**Additional file 1:** **Table S1.** Results of the pooled-taxa regressions to analyse allometry. Procrustes coordinates were regressed on the log of centroid size in MorphoJ.

## Data Availability

The datasets used and analysed during the current study are available from the corresponding author on reasonable request.
