# Supplementary material for: 3D shape analyses of extant primate and fossil hominin vertebrae support the ancestral shape hypothesis for intervertebral disc herniation
Source: BMC Evol Biol. 2019 Dec 16;19:226. doi: 10.1186/s12862-019-1550-9 (PMC6916256; doi:10.1186/s12862-019-1550-9)
Supplement: Supplementary file 1 — Additional file 1: Table S1. Results of the pooled-taxa regressions to analyse allometry. Procrustes coordinates were regressed on the log of centroid size in MorphoJ. [file 12862_2019_1550_MOESM1_ESM.docx]

Supplementary Table 1) Results of the pooled-taxa regressions to analyse allometry. Procrustes coordinates were regressed on the log of centroid size in MorphoJ.

|  | Regression Results |
| --- | --- |
| Penultimate Thoracic Vertebrae | r^2^ = 0.75, 24.6%, p<0.001 |
| Final Thoracic Vertebrae | r^2^ = 0.88, 11.6%, p<0.001 |
| First Lumbar | r^2^ = 0.87, 13.1%, p<0.001 |
